# Disruption of 5-HT_2A_ Receptor-PDZ Protein Interactions Alleviates Mechanical Hypersensitivity in Carrageenan-Induced Inflammation in Rats

**DOI:** 10.1371/journal.pone.0074661

**Published:** 2013-09-18

**Authors:** Anne-Sophie Wattiez, Xavier Pichon, Amandine Dupuis, Alejandro Hernández, Anne-Marie Privat, Youssef Aissouni, Maryse Chalus, Teresa Pelissier, Alain Eschalier, Philippe Marin, Christine Courteix

**Affiliations:** 1 Clermont Université, Université d'Auvergne, Pharmacologie Fondamentale et Clinique de la Douleur, Clermont-Ferrand, France; 2 Institut National de la Santé et de la Recherche Médicale, Unité 1107, NEURO-DOL, Clermont-Ferrand, France; 3 Laboratory of Neurobiology, Department of Biology, Faculty of Chemistry and Biology, University of Santiago of Chile, Santiago, Chile; 4 Program of Molecular and Clinical Pharmacology, Faculty of Medicine, University of Chile, Santiago, Chile; 5 Centre Hospitalier Universitaire de Clermont-Ferrand, Service de Pharmacologie, Clermont-Ferrand, France; 6 Institut de Génomique Fonctionnelle, Centre National de la Recherche Scientifique, Unité Mixte de Recherche 5203, Universités Montpellier 1 and 2, Montpellier, France; 7 Institut National de la Santé et de la Recherche Médicale, Unité 661, Montpellier, France; University of Cincinnatti, United States of America

## Abstract

Despite common pathophysiological mechanisms, inflammatory and neuropathic pain do not respond equally to the analgesic effect of antidepressants, except for selective serotonin reuptake inhibitors (SSRIs), which show a limited efficacy in both conditions. We previously demonstrated that an interfering peptide (TAT-2ASCV) disrupting the interaction between 5-HT_2A_ receptors and its associated PDZ proteins (e.g. PSD-95) reveals a 5-HT_2A_ receptor-mediated anti-hyperalgesic effect and enhances the efficacy of fluoxetine (a SSRI) in diabetic neuropathic pain conditions in rats. Here, we have examined whether the same strategy would be useful to treat inflammatory pain. Sub-chronic inflammatory pain was induced by injecting λ-carrageenan (100 µl, 2%) into the left hind paw of the rat. Mechanical hyperalgesia was assessed after acute treatment with TAT-2ASCV or/and fluoxetine (SSRI) 2.5 h after λ-carrageenan injection. Possible changes in the level of 5-HT_2A_ receptors and its associated PDZ protein PSD-95 upon inflammation induction were quantified by Western blotting in dorsal horn spinal cord. Administration of TAT-2ASCV peptide (100 ng/rat, intrathecally) but not fluoxetine (10 mg/kg, intraperitoneally) relieves mechanical hyperalgesia (paw pressure test) in inflamed rats. This anti-hyperalgesic effect involves spinal 5-HT_2A_ receptors and GABAergic interneurons as it is abolished by a 5-HT_2A_ antagonist (M100907, 150 ng/rat, intrathecally) and a GABA_A_ antagonist, (bicuculline, 3 µg/rat, intrathecally). We also found a decreased expression of 5-HT_2A_ receptors in the dorsal spinal cord of inflamed animals which could not be rescued by TAT-2ASCV injection, while the amount of PSD-95 was not affected by inflammatory pain. Finally, the coadministration of fluoxetine does not further enhance the anti-hyperalgesic effect of TAT-2ASCV peptide. This study reveals a role of the interactions between 5-HT_2A_ receptors and PDZ proteins in the pathophysiological pathways of inflammatory pain and opens new perspectives in its control thanks to molecules disrupting 5-HT_2A_ receptor/PDZ protein interactions.

## Introduction

Chronic inflammatory pain and neuropathic pain share a variety of common neuroplastic changes occurring in the spinal cord, including altered ion channel expression in dorsal root ganglion neurons, enhanced glutamate release and glutamate receptor function, as well as glial cell activation [1]. These changes are responsible for sensitization of spinal processing of afferent information, thereby causing persistent hyperalgesia and/or allodynia, which are refractory to the widely used pharmacological treatments. Despite these common central pathophysiological mechanisms, pharmacological treatment of inflammatory and neuropathic pain is different: antidepressants occupy a limited place in the therapeutic arsenal used for treating inflammatory pain [2], whereas tricyclic antidepressants (TCAs) and serotonin and noradrenaline reuptake inhibitors (SNRIs) are considered as first-line treatments of neuropathic pain [3]. The main disadvantage of antidepressants is their adverse side effects observed, for instance, in 30-100% of patients treated with TCAs [4]. In various animal pain models, such as acute inflammatory, arthritic and neuropathic pain, TCAs and dual SNRIs exhibit antinociceptive properties, whereas selective serotonin reuptake inhibitors (SSRIs) are not as efficient [5,6]. This is intriguing because serotonin (5-hydroxytryptamine, 5-HT) released from nerve terminals originating from Raphe nuclei is essential for modulation of spinal cord pain processing [7]. Moreover, the predominant inhibitory role of 5-HT on persistent pain has definitely been established in mice lacking central 5-HT neurons (Lmx1b^f/f/p^ mice): these mice exhibit enhanced persistent inflammatory pain to formalin or capsaicin injection, which is attenuated by intrathecal injection of 5-HT [8].

The 5-HT_2A_ receptor has been identified as one of the 5-HT receptors contributing to 5-HT-induced analgesia in various pain conditions. For example, central 5-HT_2A_ receptor activation inhibits C responses of wide dynamic range neurons [9] and reduces craniofacial [10] and peripheral [11] nociception induced by formalin injection or nerve ligature [11,12,13,14]. Likewise, antinociception induced by SSRIs such as fluvoxamine [15] and fluoxetine [16] as well as pain relief induced by the SNRI milnacipran [17] are mediated by 5-HT_2A_ receptor stimulation. We hypothesized that the lack of efficacy of SSRIs in inflammatory chronic pain conditions [2] might reflect alteration of 5-HT_2A_ receptor-operated signalling. This altered receptor functionality might result from abnormal receptor interactions with regulatory proteins, in line with previous findings indicating that 5-HT_2A_ receptors associate with multiple intracellular proteins, which are essential for the regulation of their functional status [18,19]. These include PSD‑95/Disc Large/Zonula occludens-1 (PDZ) domain containing proteins of the membrane-associated guanylate kinase (MAGUK) family [20]. Consistent with this hypothesis, we previously demonstrated that disrupting the interactions between spinal 5-HT_2A_ receptors and associated PDZ proteins by an interfering peptide able to transduce into spinal neurons *in vivo* after intrathecal injection, inhibited thermal and mechanical hyperalgesia and enhanced fluoxetine-induced analgesia [21]. The peptide comprising the nine C-terminal residues of the 5-HT_2A_ receptor and fused with the transduction domain of HIV type 1 Tat protein (amino acid sequence YGRKKRRQRRRTVNEKVSC, TAT-2ASCV) was also shown to prevent association between the receptor and its MAGUK partners PSD-95 and SAP97 *in vitro* [21]. As previous studies have demonstrated a role of MAGUKs in chronic inflammatory pain [22,23,24], we wondered whether association of spinal 5-HT_2A_ receptors with PDZ proteins might also influence regulation of inflammatory pain and, accordingly, whether the same peptidyl mimetic strategy that competes for interactions between the receptor’s PDZ-binding motif and target PDZ proteins would be useful to treat inflammatory pain. To address this issue, we have examined the effect of spinal injection of the TAT-2ASCV peptide toward mechanical hypersensitivity induced by carrageenan in rats. We also investigated the impact of TAT‑2ASCV peptide administration on the response to fluoxetine in carrageenan-induced inflammatory pain.

## Materials and Methods

### Ethics statement

The ethical guidelines for investigation of experimental pain in conscious animals [25] were respected, and the experimental protocol was approved by the Local Ethics Committee for Animal Experimentation (CEMEAA authorization No. CE-07-08). Great care was taken to avoid or minimize discomfort of the animals.

### Animals and pain model

Male Sprague-Dawley rats (Charles River, Cléon, France) weighing 226-250 g were housed in standard laboratory conditions under a 12-h light-dark schedule with free access to food and water, for one week before starting the experiments.

Inflammatory pain was induced by injecting 100 µl of a 2% solution of λ-carrageenan into the left hind paw of the rat [26]. Carrageenan caused visible redness and pronounced swelling that was well developed 2 h after the injection, maximal between 2 to 4 h after the injection, and persisted for more than 48 h [27]. The animals were tested 2.5 h after the λ-carrageenan injection.

### Behavioral testing

Hypersensitivity to mechanical stimuli was assessed using an analgesy-meter (Ugo Basile, Bioseb®, France) by applying a linearly increasing mechanical force to the dorsum of the left hind paw until a squeak was obtained. Because this test involves animal handling, the experimenter got the rats used to being handled as follows: 3 days before the experiment, rats were held by the experimenter for 20 s in the same way that for the paw pressure test without escaping. On the day of the experiment, rats were again handled 3 times by the experimenter for 20 s and, simultaneously, the Ugo-basile analgesy-meter was started started (without applying any pressure to the rat’s hind paw) to get the rat used to the noise of the apparatus. No rats showed aversive reactions during handling. Then, the paw of the rat was placed under the tip, and the pressure progressively applied until the rat vocalized. Three to four measures were performed at 10 min intervals in order to obtain two consecutive values that differed no more than 10%. The vocalization threshold was defined as the mean score of two measures, expressed in arbitrary units (A.U.), one A.U. corresponding to a 10 g force. The maximal pressure (cut-off) applied was 75 A.U (i.e. 750 g).

### Injections

TAT-2ASCV, NaCl, M100907 and bicuculline were intrathecally (i.t.) injected in a volume of 10 µl in the subarachnoid space between L5 and L6 using a 25 G x 1/2 inch needle and a 25 µl Hamilton syringe as described by Mestre et al. (1994) [28]. Intrathecal injection is stressful for rats and, according to IASP recommendation, requires anaesthesia. Thus, before injection, rats were slightly anaesthetized with volatile isoflurane (3.5%) and the needle was inserted through skin and muscles, perpendicularly to the spinal cord. A rapid reflex movement of the tail indicated that the needle had reached the right space, and the drug was injected slowly to avoid any reflux. Rats recovered 5 minutes after the removal from the anaesthesia chamber. The eventual effect of anaesthesia could be observed in the control groups (vehicle injection). For intrathecal co-administrations, each drug was injected separately (10 µl each).

Fluoxetine and HPMC were intraperitoneally (i.p.) administered in a volume of 5 ml/kg.

### Drugs and experimental design

Bicuculline (GABA_A_ receptor antagonist), λ-carrageenan and M100907 (5-HT_2A_ receptor antagonist) were purchased from Sigma-Aldrich (Saint-Quentin Fallavier, France). Drugs were dissolved in physiological saline (NaCl, 0.9%), except M100907 and bicuculline, dissolved in 25% dimethyl sulfoxide (DMSO), λ-carrageenan, in distilled water and fluoxetine, in 0.25% hydroxy-propyl-methyl-cellulose (HPMC).

Synthetic peptides (> 95% purity) were purchased from Eurogentec (Seraing, Belgium). The sequences of TAT-2ASCV and TAT-2ASCA (TAT-2ASCV in which the C-terminal valine was substituted by an alanine) peptides were as follows: TAT-2ASCV, YGRKKRRQRRRTVNEKVSCV; TAT-2ASCA, YGRKKRRQRRRTVNEKVSCA.

Drugs were prepared just before the injections. All experiments were performed by the same experimenter blinded to the treatment. Treatments were randomized and administered according to the method of blocks in order to assess their effect in the same conditions. Different animals were used in each experiment.

Baseline mechanical sensitivity was determined before and 2.5 h after carrageenan injection. At that point, rats were considered as hyperalgesic and included in the study if their vocalization thresholds in response to paw-pressure were reduced by more than 15% of the value obtained prior to the induction of the inflammation. Then, for each experimental series, hyperalgesic rats were randomly assigned into treatment groups. Rats received an i.t. administration of either vehicle (NaCl 0.9%, 10 µl/rat), or TAT-2ASCV (30 or 100 ng/rat) or TAT-2ASCA (100 ng/rat) or a co-administration of TAT-2ASCV (100 ng/rat, i.t.) and either vehicle or M100907 (150 ng/rat, i.t.), or bicuculline (3 µg/rat,i.t.), or fluoxetine (10 mg/kg i.p.). The vehicle was 25% DMSO (10 µl/rat, i.t.) for bicuculline and M100907, and 0.25% HPMC (5 ml/kg i.p.) for fluoxetine.

### Preparation of spinal cord extracts

Carrageenan-treated hyperalgesic and NaCl-treated rats were sacrificed by decapitation 2.5 h after the λ-carrageenan or NaCl injection. Carrageenan-treated hyperalgesic rats were sacrificed 0.5 h after i.t. TAT-2ASCV or vehicle injection, corresponding to the delay to obtain the maximal analgesic effect. Lumbar enlargements of the spinal cord dorsal horn were rapidly removed and the ipsilateral side to carrageenan or vehicle injection homogenized on ice with lysis buffer containing Tris-HCl, 50 mM, pH 7.4; EDTA, 0.5 mM; 3-[(3-cholamidopropyl) dimethylammonio] propanesulfonate (CHAPS, 1.3% wt/vol), and a protease inhibitor cocktail (Roche Diagnostics, Mannheim, Germany). Samples were centrifuged for 1 h at 10,000 x g and the supernatants containing CHAPS-solubilized proteins were collected.

### Western blotting

Proteins resolved on 10% polyacrylamide gels were transferred electrophoretically to nitrocellulose membranes (Hybond-C; GE Healthcare) and stained with Ponceau red, to assess transfer of equal amounts of proteins in each lane. Membranes were blocked with 5% non-fat dry milk diluted in Tris-Buffered Saline Tween-20 and incubated successively with the primary antibody (anti-5-HT_2A_ receptor mouse IgG_1_, 1:500 (PharMingen, San Diego, CA, USA); rabbit polyclonal 5-HT_2A_ receptor antibody, 1:200 (Santa-Cruz Biotechnology, Heidelberg, Germany); anti-PSD-95 mouse IgG_2A_, 1:50,000 (clone K28/43) (Upstate Biotechnology, Charlottesville, NC, USA); mouse anti-pan-actin, 1:2,000 (NeoMarquers, Fremont, CA, USA) overnight at 4°C and with a horse-radish peroxidase-conjugated anti-mouse antibody (1:3000; GE Healthcare) for 1 h at room temperature. Immunoreactivity was detected with an enhanced chemiluminescence method (ECL^TM^ detection reagent, GE Healthcare).

### Data analysis

Vocalization thresholds were expressed as mean ± standard error (S.E.) of raw data in arbitrary units (A.U.). Behavioral data were examined using a two-way analysis of variance (ANOVA, repeated measures). When significant, the ANOVA was followed by a Student-Newman-Keuls test in order to compare the different groups at the same time and to analyze the time-course effect of treatments. The area under the time-course (0-60 min) curve (A.U.C.) of vocalization threshold (VT) variations (individual values at time T = (thresholds at time T – thresholds obtained before drug treatment)) was calculated by the trapezoidal rule and expressed as mean ± S.E. (in A.U. x minutes). A one-way analysis of variance (ANOVA) was used to compare A.U.C. and when significant, was followed by a Bonferroni test. The significance level was set at P<0.05. Statistical analyses were run using SigmaStat 3.10/Systat Software, Inc.

## Results

### Effect of the injection of λ-carrageenan in the left hind paw on mechanical sensitivity

Before the injection of carrageenan, rats did not display any difference in their vocalization thresholds. Mechanical hypersensitivity developed within 2.5 hours after carrageenan injection and persisted for 90 minutes. Vocalization thresholds to paw pressure displayed a significant decrease (more than 15% reduction) compared with those measured before carrageenan (38.4 ± 0.6 *vs.* 24.0 ± 0.4A.U., respectively). All rats (n = 96) were considered hyperalgesic and were included in the study.

### Effect of disrupting interactions between 5-HT_2A_ receptors and PDZ proteins on inflammatory pain-induced hyperalgesia

**Figure 1:**
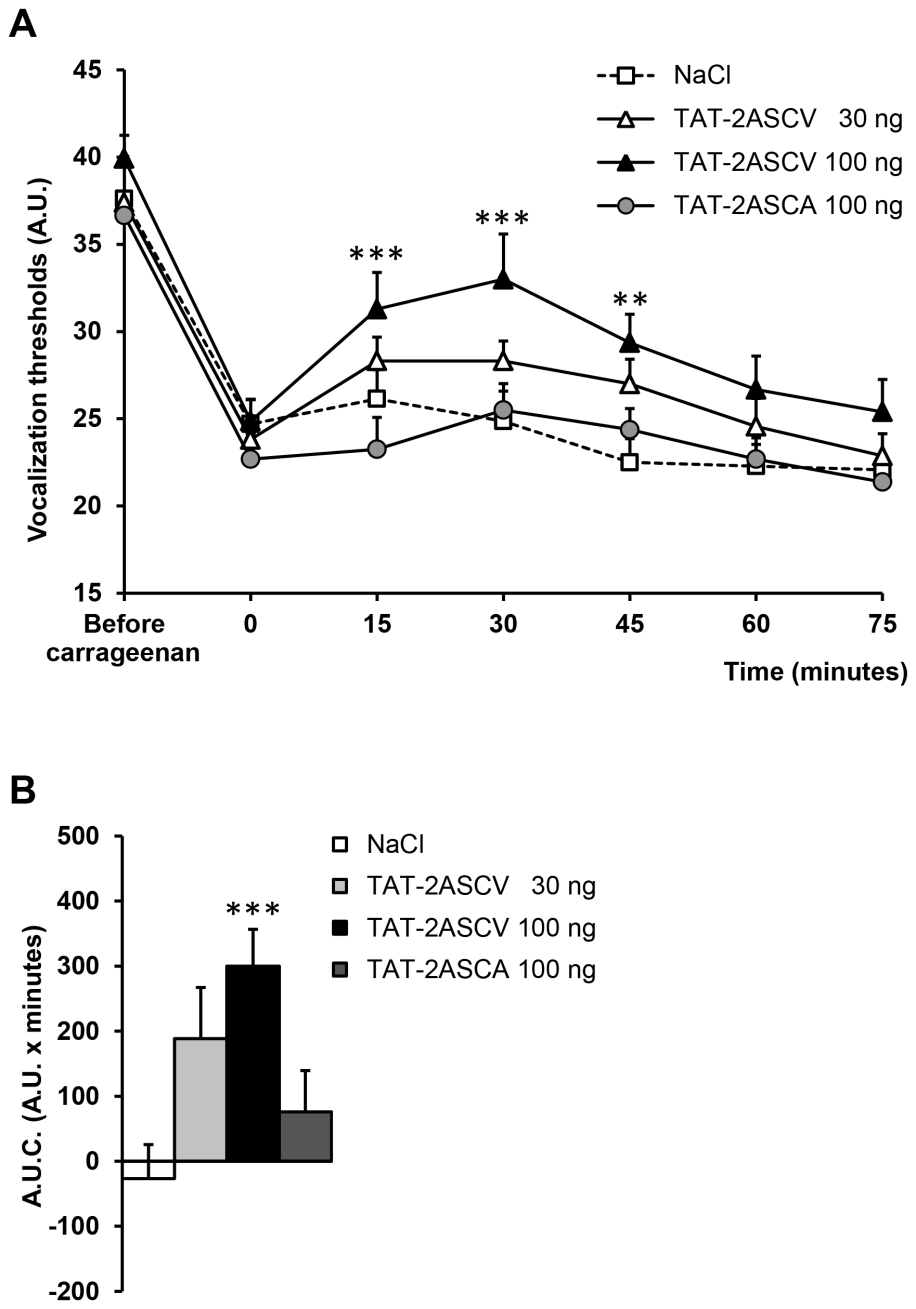
Disruption of 5-HT_2A_ receptor/PDZ protein interactions produces anti-hyperalgesic effects in carrageenan-injected rats. Carrageenan-treated hyperalgesic rats were injected with either TAT-2ASCV (30, 100 ng/rat) or TAT-2ASCA (100 ng /rat) or NaCl (10 µl/rat). The vocalization thresholds to paw-pressure were measured up to 75 min after the injection (A). **P<0.01 and ***P<0.001, compared with values measured before the peptide/NaCl injection (time 0). (B) Area under the time-course (0-60 minutes) of vocalization threshold variations. ***P<0.001 vs. NaCl-treated group.

Intrathecal injection of NaCl or TAT-2ASCV 30 ng/rat had no effect on mechanical hyperalgesia (Figure 1A, B). Only TAT-2ASCV at the dose of 100 ng/rat significantly increased vocalization thresholds as shown by measuring the time course of vocalization thresholds. The global effect of the peptide measured by the area under the curves (A.U.C) of the threshold variations (Figure 1A, B), confirmed that only the 100 ng dose induces a significant effect. A maximal anti-hyperalgesic effect was observed 30 min after the injection and lasted 45 minutes (Figure 1A). In contrast, the TAT-2ASCA peptide (100 ng/rat, i.t.), which did not inhibit the interactions between the 5-HT_2A_ receptor and its PDZ partners [21], did not exert any anti-hyperalgesic action against mechanical stimulation, as shown by the lack of significant increase of paw-pressure-induced vocalization thresholds (Figure 1A, B).

### Effect of the co-administration of the peptide TAT-2ASCV with 5-HT_2A_ and GABA_A_ receptor antagonists on inflammatory pain-induced hyperalgesia

**Figure 2:**
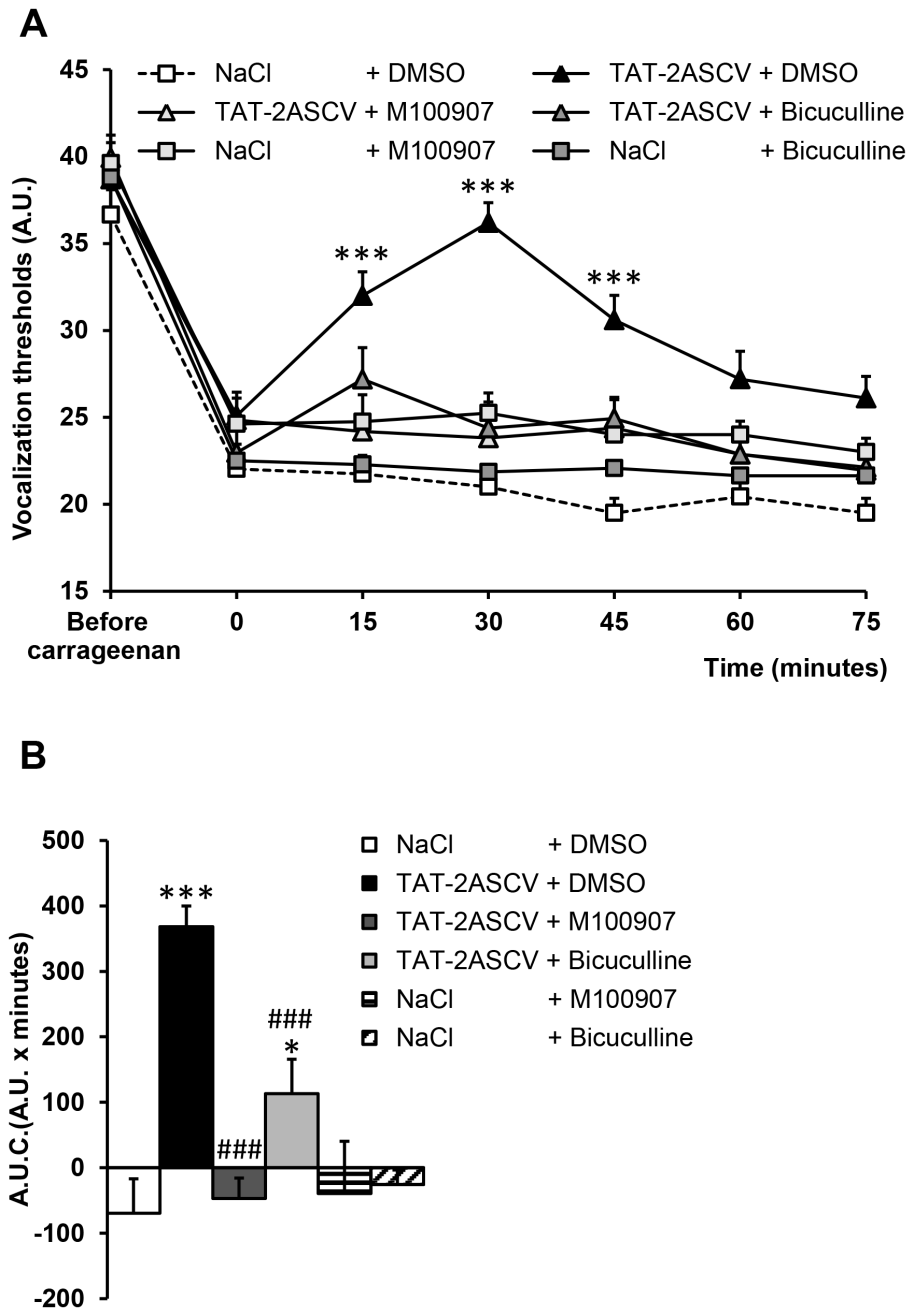
Inhibition of the anti-hyperalgesic effect of TAT-2ASCV by M100907 or bicuculline in carrageenan-injected rats. Carrageenan-treated hyperalgesic rats were injected with M100907 (150 ng/rat) or bicuculline (3 µg/rat) in the absence or presence of the TAT-2ASCV peptide (100 ng/rat, i.t.). The vocalization thresholds to paw-pressure were measured up to 75 min after the injections (A). *P<0.05 and ***P<0.001, compared with values measured before the injections (time 0). (B) Area under the time-course (0-60 minutes) of vocalization threshold variations. *P< 0.05 and ***P<0.001 *vs*. NaCl + DMSO-treated group, ^# # #^ P<0.001 *vs*. TAT-2ASCV + DMSO-treated group.

The anti-hyperalgesic effect of TAT-2ASCV (100 ng/rat) was abolished by the co-administration of M100907 (150 ng/rat, i.t.), indicating that is was mediated by 5-HT_2A_ receptors (Figure 2A, B). The co-administration of bicuculline (3 µg/rat, i.t.) likewise suppressed the anti-hyperalgesic effect of TAT-2ASCV, indicative of implication of GABAergic interneurons in the analgesic effect induced by 5-HT_2A_ receptor activation in this inflammatory pain model (Figure 2A, B).

### Effect of λ-carrageenan injection upon 5-HT_2A_ receptor and PSD-95 expression in spinal dorsal horn.

**Figure 3:**
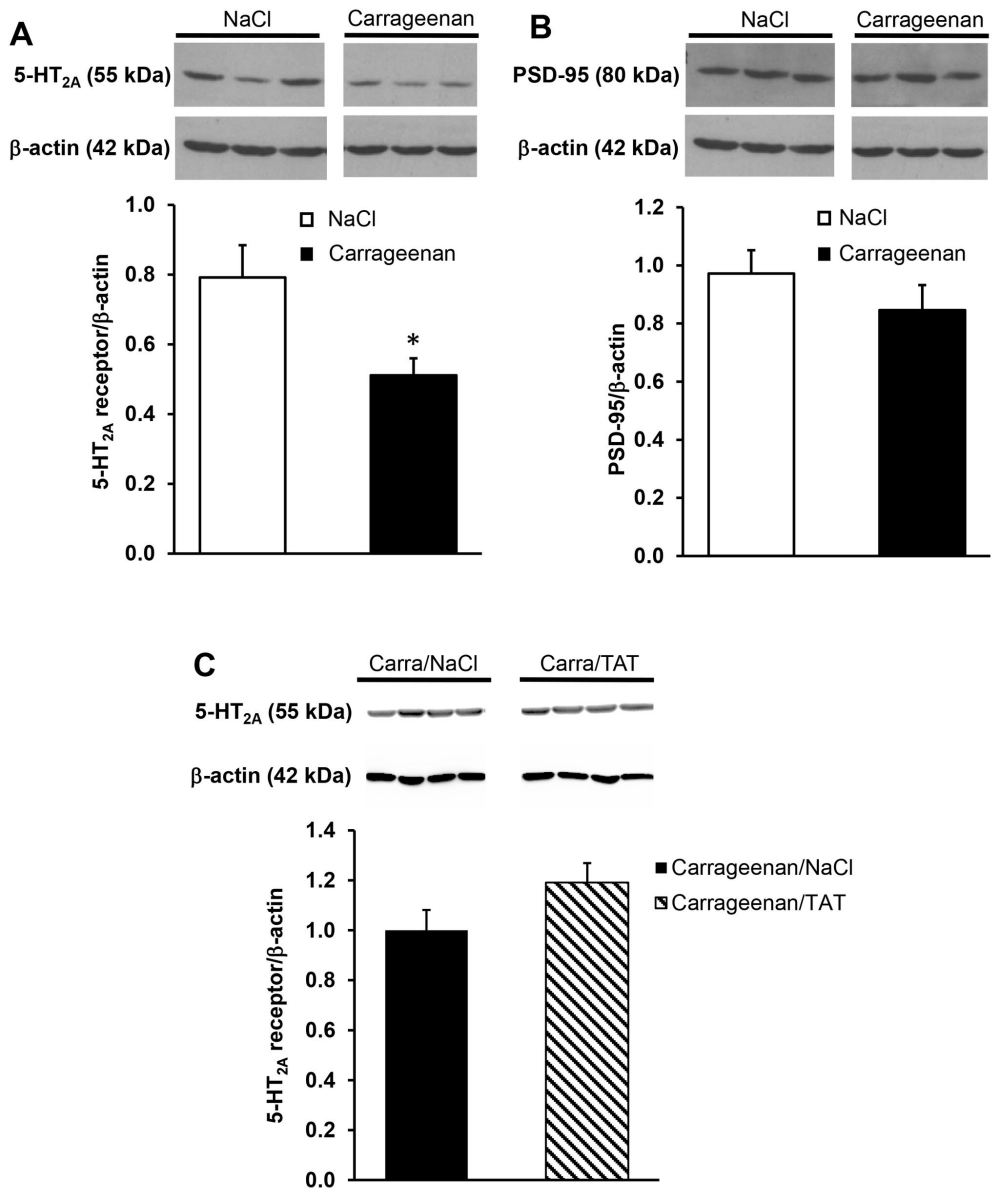
Decreased expression of 5-HT_2A_ receptors but not of PSD-95 in spinal cord of carrageenan-injected rats. Expression of 5-HT_2A_ receptors (A) and PSD-95 (B) in spinal cord extracts ipsilateral to paw injection of NaCl- or carrageenan-treated rats were analyzed by Western blotting. Representative gels obtained from 3 NaCl-treated rats, 3 carrageenan-treated rats, 4 carrageenan-NaCl-treated rats and 4 carrageenan-TAT-2ASCV-treated rats are illustrated in A, B and C respectively. Data, expressed in A.U. normalized to beta-actin, are means ± SEM of values obtained in 9 NaCl-treated rats, 9 carrageenan-treated rats, 4 carrageenan-NaCl-treated rats and 4 carrageenan-TAT-2ASCV-treated rats. *P< 0.05 *vs*. corresponding NaCl-treated group.

Western-blotting experiments indicated a decreased expression of 5-HT_2A_ receptors in carrageenan-injected rats, compared with NaCl-injected rats (36 ± 6% decrease, Figure 3A). Previous experiments indicated an increase in PSD-95 expression in dorsal spinal cord of diabetic neuropathic rats, compared with healthy rats [21]. In contrast, we found that the expression of PSD-95 in dorsal spinal cord was not affected in conditions of inflammatory pain (CARRA, Figure 3B). Nonetheless, the above observation shows a modification of the relative proportion of the 5-HT_2A_ receptor and its major PDZ partner PSD-95 in the dorsal spinal cord upon induction of inflammatory pain. Intrathecal injection of TAT-2ASCV (100 ng/rat) failed to rescue the decrease of 5-HT_2A_ receptors induced by peripheral inflammation (Figure 3C).

### Effect of the co-administration of TAT-2ASCV with fluoxetine on inflammatory pain-induced hyperalgesia.

**Figure 4:**
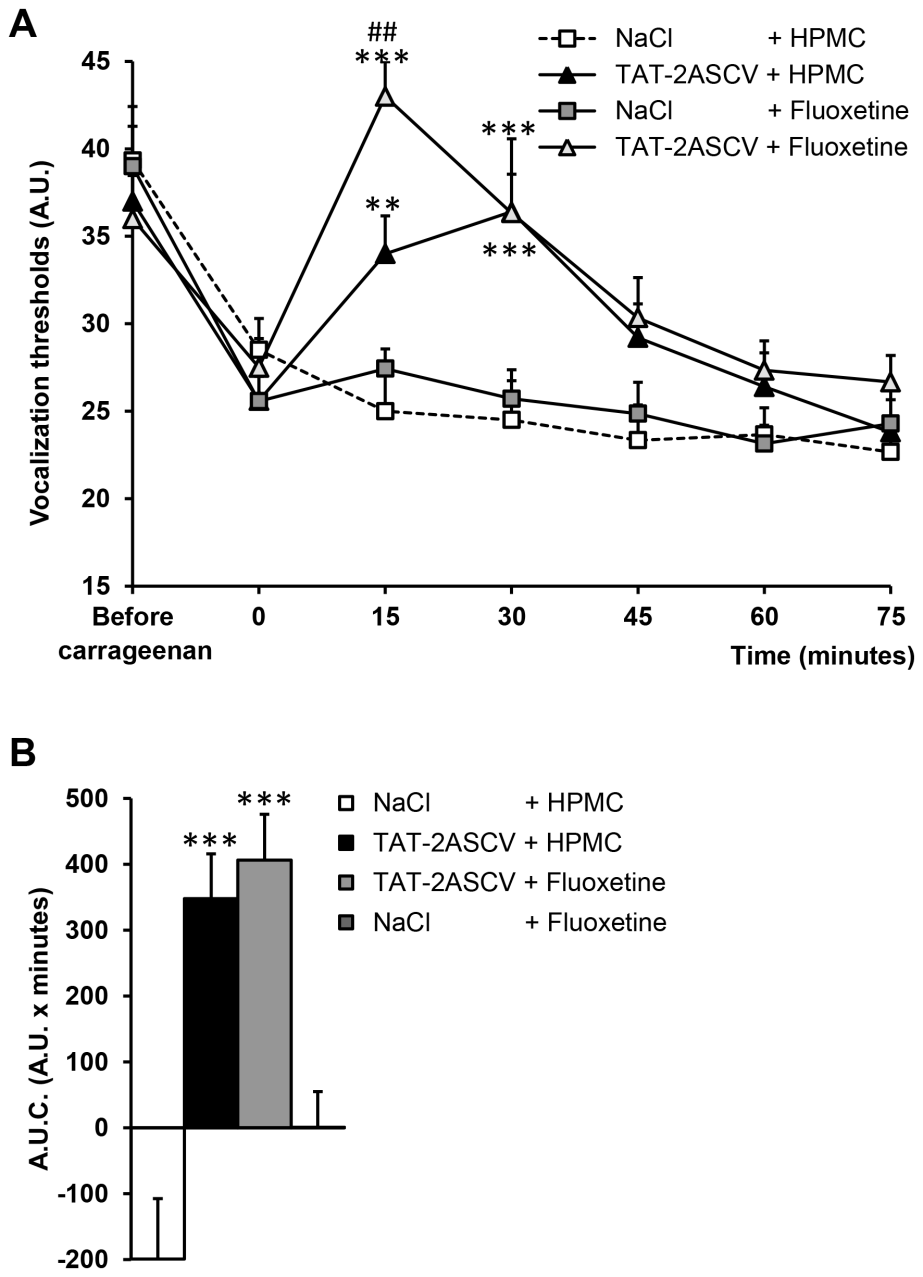
The disruption of 5-HT_2A_ receptor/PDZ protein interactions fails to enhance fluoxetine efficacy. (A) Carrageenan-treated hyperalgesic rats received an injection of either HPMC (5 ml/kg i.p.) or fluoxetine (10 mg/kg i.p.). They were then immediately injected with either NaCl (10 µl/rat, i.t.) or TAT-2ASCV peptide (100 ng/rat, i.t.). The vocalization thresholds to paw-pressure were measured up to 75 min after the injections. *P<0.05 and **P<0.01, compared with values measured before the injections (time 0). (B) Area under the time-course (0-60 minutes) of vocalization threshold variations. *P< 0.05 and **P<0.01 *vs*. TAT-2ASCV (100 ng/rat)-treated group.

Administration of fluoxetine (10 mg/kg i.p.) did not increase vocalization thresholds in carrageenan-hyperalgesic rats at any times tested (Figure 4A). Administration of fluoxetine combined with the intrathecal injection of TAT-2ASCV (100 ng/rat, i.t.) produced an anti-hyperalgesic effect (maximal VT at 15 min: 43.0 ± 2.0 A.U; A.U.C: 406 ± 70 A.U. x min), which was not significantly different from that induced by the peptide alone (maximal VT at 30 min: 36.4 ± 4.2 A.U ; A.U.C. : 348 ± 68 A.U. x min) (Figure 4A, B).

## Discussion

As previously reported in diabetic neuropathic rats, the present study shows that (i) disrupting interactions between spinal 5-HT_2A_ receptors and associated PDZ proteins by intrathecal injection of the cell-penetrating peptidyl mimetic of the receptor C-terminus (TAT‑2ASCV), dose-dependently reduces mechanical hyperalgesia in rats with carrageenan-induced sub-chronic inflammation and (ii) that this effect is likely mediated by activation of 5-HT_2A_ receptors located on GABAergic interneurons of the spinal cord. Indeed, intrathecal delivery of either the 5-HT_2A_ receptor antagonist M100907, or the GABA_A_ receptor antagonist bicuculline suppressed the anti-hyperalgesic effect of TAT-2ASCV (also delivered intrathecally). Recently, it has been reported that GABA released from spinal GABAergic interneurons upon 5-HT_2A_ receptor activation by endogenous 5-HT in turn inhibits the spinal nociceptive pathway [29]. The expression of 5-HT_2A_ receptor in GABAergic neurons of the spinal dorsal horn has clearly been established [30], further supporting a role of 5-HT_2A_ receptors in GABA release in the spinal cord.

It has been shown previously that the spinal injection of TAT-2ASCV suppresses thermal and mechanical hypersensitivity in diabetic neuropathic rats [21]. However, one could not predict whether the same strategy would also be effective on inflammatory pain, because inflammatory pain and neuropathic pain exhibit different susceptibility to drugs (non-steroidal anti-inflammatory drugs *vs.* antidepressants, respectively). Carrageenan-induced hyperalgesia arises as a consequence of the activation and sensitization of primary nociceptive afferents, followed by central sensitization of dorsal horn neurons in the spinal cord and a concomitant expansion of their receptive fields and plasticity of their neuronal connections (see e.g. [31,32]). We previously demonstrated that TAT-2ASCV can restore 5-HT_2A_ receptor functionality by specifically inhibiting 5-HT_2A_ receptor-PDZ protein interactions. These findings together with the present observations that TAT-2ASCV significantly reduces mechanical hyperalgesia *via* a mechanisms dependent on 5-HT_2A_ receptor stimulation in carrageenan-treated rats, suggest that inflammatory hyperalgesia might also result from either a loss of responsiveness of 5-HT_2A_ receptors to endogenous serotonin and/or a down-regulation of these receptors. Interestingly, we found that the expression level of 5-HT_2A_ receptors was significantly decreased in the spinal cord of carrageenan-induced hyperalgesic rats, suggesting that 5-HT_2A_ receptor down-regulation might contribute, at least in part, to hyperalgesia elicited by carrageenan injection. The decrease in 5-HT_2A_ receptor expression seems to distinguish inflammatory pain from neuropathic pain [21]. The mechanism underlying carrageenan-induced 5-HT_2A_ receptor down-regulation remains to be elucidated. A decreased expression of the overall 5-HT_2A_ receptor population within a 2.5 h period, as detected by Western-blotting, could result from an increased lysosomal degradation following receptor ubiquitination and endocytosis, since 5-HT_2A_ receptors are known to be recycled in about 2.5 hours [33]. In contrast to our study, Zhang et al. demonstrated that the expression of 5-HT_2A_ receptor mRNA in spinal cord [34] as well as the release of 5-HT and its major metabolite 5-HIAA [35] were markedly increased following carrageenan inflammation. The reasons for the discrepancy between their findings and the present observation are not clear. The same concentration of carrageenan was injected in both studies, but the volume of carrageenan solution injected in the study of Zhang et al. was 50% higher than that used in the present study. Anyway, the study of Zhang et al. indicated a role of 5-HT_2A_ receptors in suppression of inflammatory pain, consistent with the present observations and support the involvement of endogenous 5-HT in the effect of the TAT-2ASCV peptide. Interestingly, whereas TAT-2ASCV showed analgesic efficacy on inflammatory pain, it failed to resolve 5-HT_2A_ receptor down-regulation. Whether the disruption of 5-HT_2A_ receptor/ PSD-95 protein interactions regulates signal transduction and/or intracellular trafficking of 5-HT_2A_ receptors as previously reported [36], remains to be determined. This suggests that 5-HT_2A_ receptor functional activity rather than total number of 5-HT_2A_ receptors should be targeted to improve endogenous pain control.

We detected no change in the expression level of PSD-95 in spinal dorsal horn of carrageenan-injected rats, compared with vehicle-treated animals whereas an increased level of PSD-95 was observed following STZ-induced diabetic neuropathy [21]. Accordingly, down-regulation of 5-HT_2A_ receptors resulted in a ~26% increase in PSD-95 protein /5-HT_2A_ receptor ratio in dorsal spinal cord of carrageenan-treated rats. These observations suggest that hyperalgesia following tissue and nerve injury may result from different mechanisms (i.e. decrease in 5-HT_2A_ receptor expression or increase in PSD-95 ptotein expression) converging *in fine* to an imbalance of PSD-95 protein/5-HT_2A_ receptor ratio and consequently to a decrease of receptor functional activity and endogenous pain control. The role of PSD-95 in the generation and the maintenance of inflammatory pain in rats is well documented [22,23,24]. Moreover, PSD-95 might contribute to the effect of drugs favouring 5-HT transmission such as SSRIs in neuropathic pain [21]. Whether PSD-95 contributes to hyperalgesia in carrageenan-treated rats and whether the effect of TAT-2ASCV results from disruption of 5-HT_2A_ receptor/PSD-95 interaction remains to be established. PSD-95 is known to interact with other receptors known to control pain transmission (e.g. NMDA receptors). Accordingly, it is unlikely that spinal PSD-95 knockdown using shRNA would allow definite conclusion regarding the role of 5-HT_2A_ receptor/PSD-95 interaction *vs.* for instance NMDA receptor/PSD-95 interaction, in carrageenan-elicited inflammatory pain.

Finally, we report that the SSRI fluoxetine, which was poorly effective in painful rheumatic patients [37], fails to reduce carrageenan-induced inflammatory pain in rats. The dose used (10 mg/kg) was already shown to increase the tail electric shock-induced vocalization threshold in carrageenan-injected rats [38] but not thermal hyperalgesia and mechanical allodynia [39]. The co-administration of the TAT-2ASCV with the SSRI did not reveal the analgesic efficacy of the antidepressant, indicating that the 100 ng/rat dose of TAT‑2A-SCV was sufficient to enhance the responsiveness of 100% of the 5-HT_2A_ receptors, and thereby to induce a maximal anti-hyperalgesic effect. This contrasts with previous observations in diabetic neuropathic rats where the peptide strongly potentiated the effect of fluoxetine [21] and suggests that 5-HT_2A_ receptor/PDZ protein interactions might not be critical for the resistance of inflammatory pain to SSRIs.

## Conclusions

This study demonstrates that disrupting 5-HT_2A_ receptor/PDZ protein interactions using a peptidyl mimetic strategy abolishes carrageenan-induced inflammatory mechanical hypersensitivity by recovering 5-HT_2A_ receptor functionality. Recently, the use of quinoline ligands has shown to specifically inhibit 5-HT_2A_ receptor/ PSD-95 interactions and to suppress neuropathic pain in rats [40]. These results, together with the present findings, point out the relevance of strategies blocking protein/protein interactions to manage chronic and sub-chronic pain, with presumably less pronounced side effects than classical approaches based on receptor agonists or antagonists.
